# Supplementary material for: Modelling liver cancer microenvironment using a novel 3D culture system
Source: Sci Rep. 2022 May 14;12:8003. doi: 10.1038/s41598-022-11641-7 (PMC9107483; doi:10.1038/s41598-022-11641-7)
Supplement: Supplementary file 1 — Supplementary Information 1. [file 41598_2022_11641_MOESM1_ESM.docx]

**Supplementary information**

Ala’a Al-Hrout^1,2^, Karla Cervantes-Gracia^1^, Richard Chahwan^1*^, Amr Amin^2^ *

^1^ Institute of Experimental Immunology, University of Zurich, 8057 Zurich, Switzerland

^2^ Biology Department, College of Science, UAE University, P.O. Box 15551, Al-Ain, UAE

*Corresponding to: [a.amin@uaeu.ac.ae](mailto:a.amin@uaeu.ac.ae) , Biology Department, UAE University, P.O. Box 15551, Al-Ain, UAE

*Corresponding to: [chahwan@immunology.uzh.ch](mailto:chahwan@immunology.uzh.ch), Institute of Experimental Immunology, University of Zurich, 8057 Zurich, Switzerland

**SUPPLEMENTARY INFORMATION**

**Supplementary data**: supplementary figures

**Table S1**: Canonical pathways list.

**Table S2**: miRNA gene interaction list.

**Supplementary File 1:** The interactive diagram of downregulated genes can be accessed on Figshare ([10.6084/m9.figshare.19519438](https://doi.org/10.6084/m9.figshare.19519438))

**Supplementary File 2:** The interactive diagram of upregulated genes can be accessed on Figshare ([10.6084/m9.figshare.19519441](https://doi.org/10.6084/m9.figshare.19519441))

**Supplementary Figures**

**Figure S1.** Supporting material for Fig. 1. a) Representative images of spheroids generated between day 2-5. Scale bar= 50 µm. b) Size range of day 2-5 spheroids in µm. Data represented as mean ± SD. c) Uncropped blots of Fig. 1. Experimental conditions are denoted above the graph. L signify protein ladder. All shown blot edges are a result of manually cutting the membrane prior to immunoblotting. The only exception is the dotted line shown on the blot in the far right; in that case the membrane was cropped since the lanes beyond those shown are for another unrelated experiment. When the background has a low contrast, the manually cut edges are shown in grey square edge.


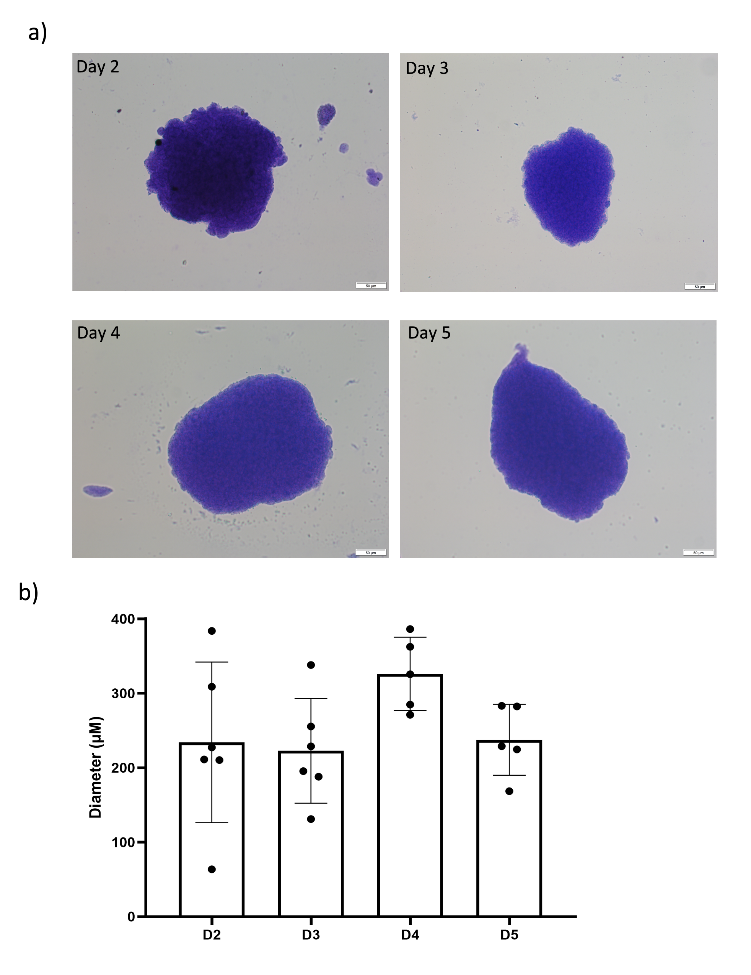


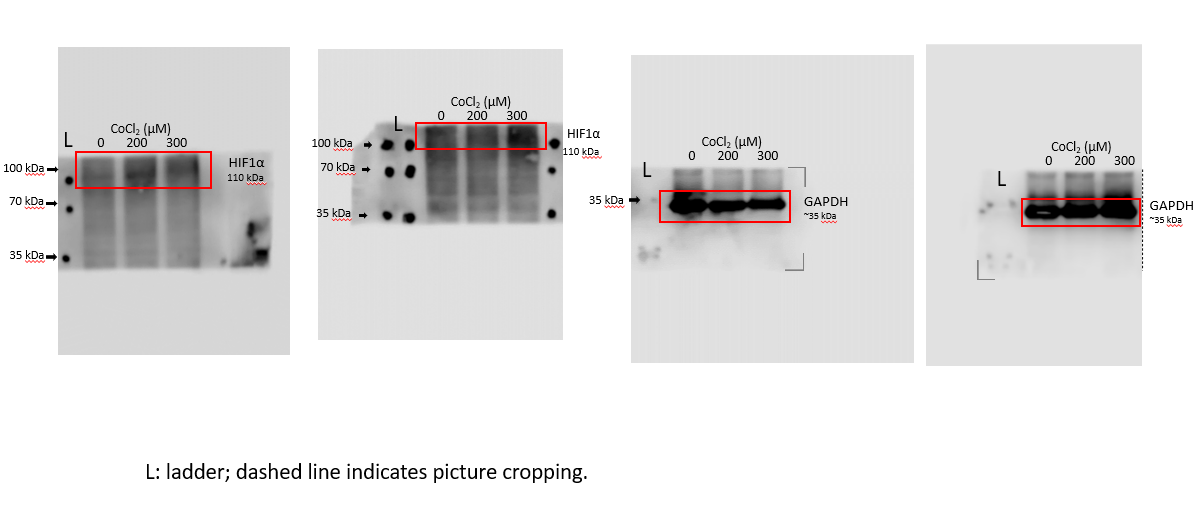


c)

**Figure S2.** Supporting material for Fig. 4. a) Schematic design of experimental outline (created with BioRender.com). b) levels of different secreted factors in 3D mono- and co-cultures analyzed by antibody arrays.


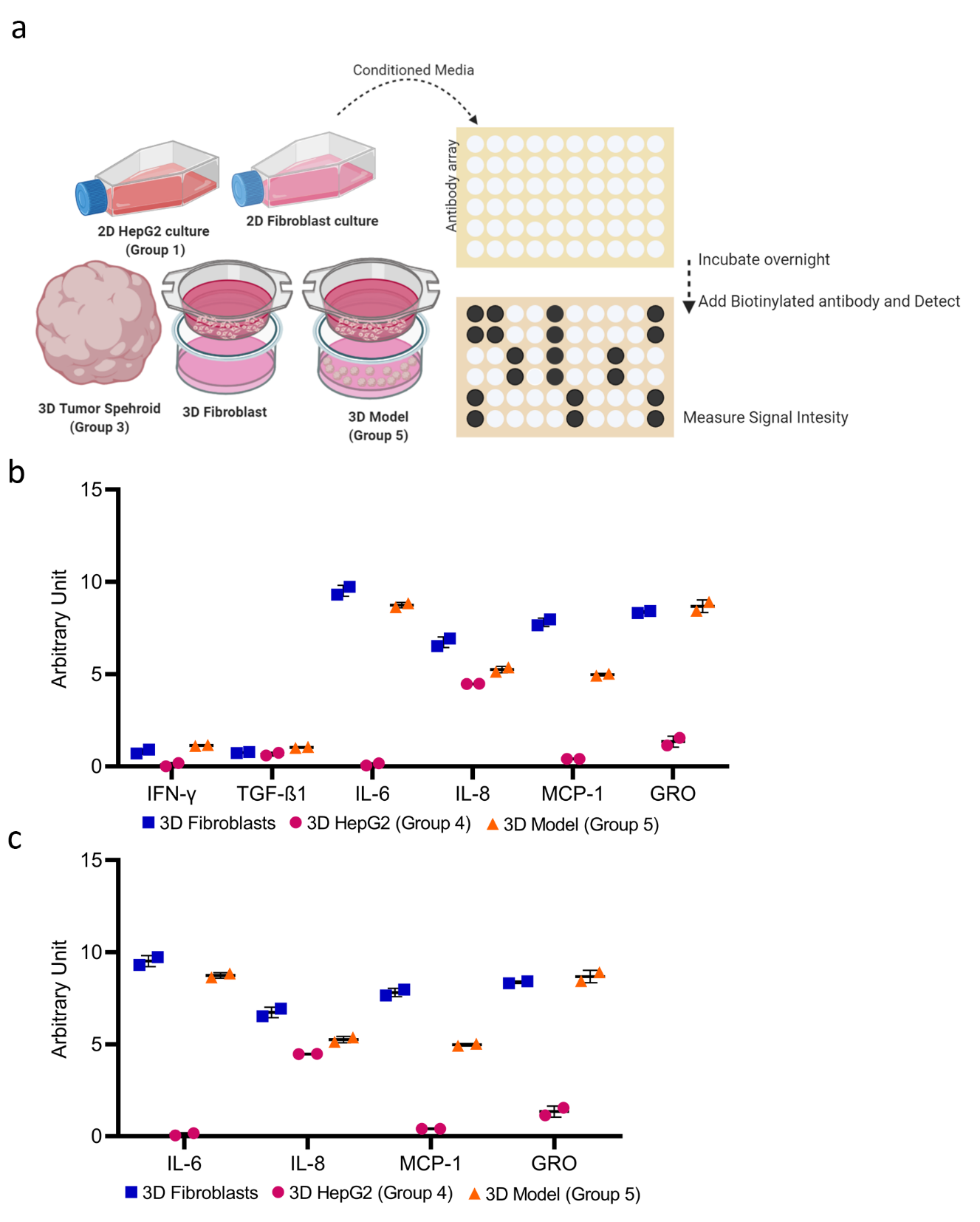


**Figure S3.** Uncropped blots of Fig. 4. Experimental conditions are denoted above the graph. L signify protein ladder. All shown blot edges are a result of manually cutting the membrane prior to immunoblotting. When the background has a low contrast, the edges are shown in grey square edge.


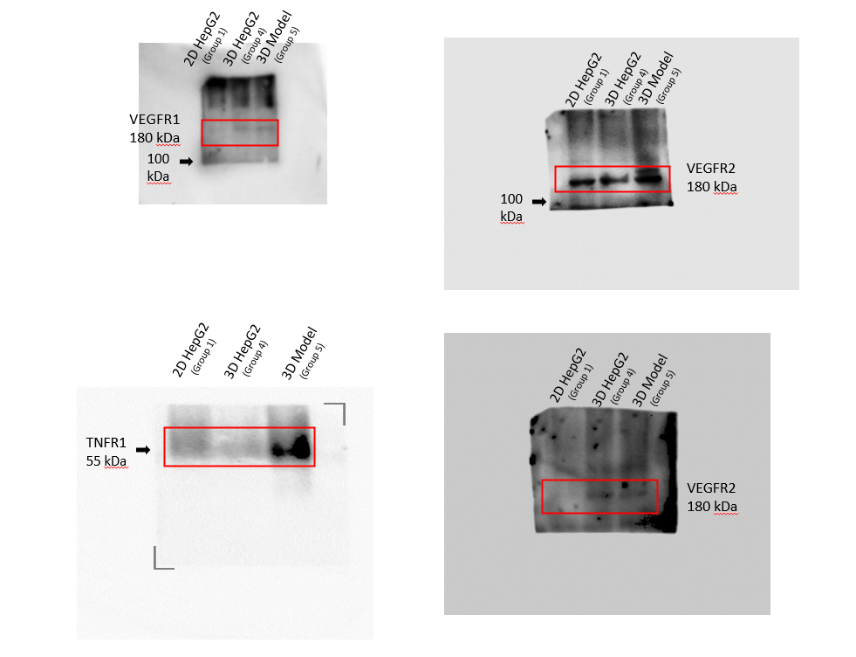


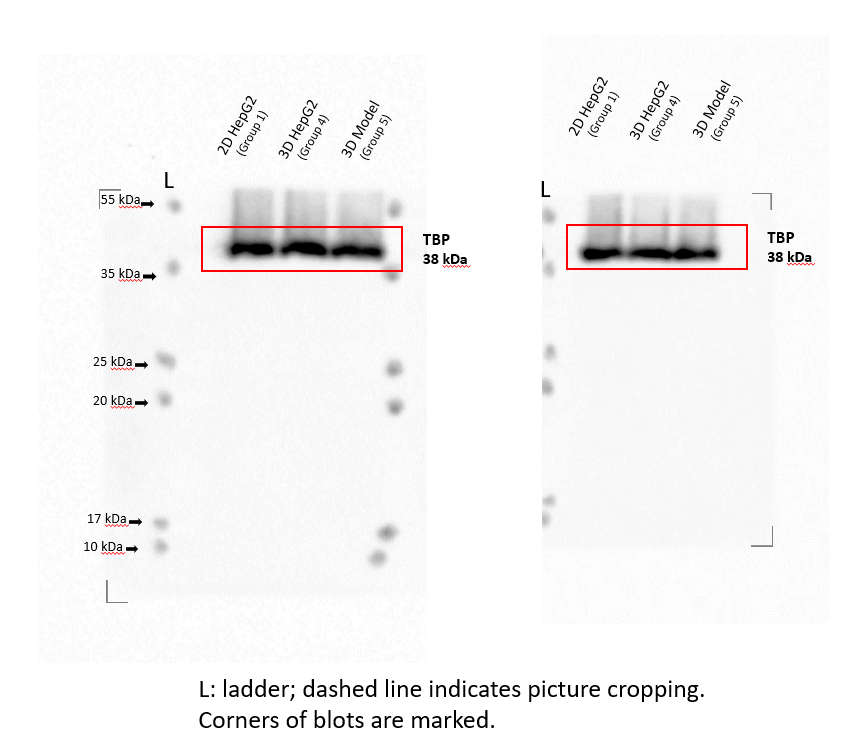


**Figure S4.** **Customized Insulin signaling pathway map of Group 5 DEGs.** Insulin signaling pathway identified as significant through ClueGO/CluePedia pathway term clustering analyzed and customized through Pathvisio. Red boxes: up regulated genes; green boxes: down regulated genes; gray boxes: genes not found within the DEGs. Color intensity resembles the deregulated status of the gene from the LogFC scale (between a 1 to -1 LogFC ). A clear up-regulated trend was identified that resembles the activation of the pathway and the main genes involved within this mechanism. This customized pathway map delineates the line of significance from G5 Insulin signaling pathway. These results back-up and *validate ClueGO/CluePedia analysis. Yellow and blue stars highlight the genes/proteins that were further validated either through antibody arrays or qPCR respectively.*

**
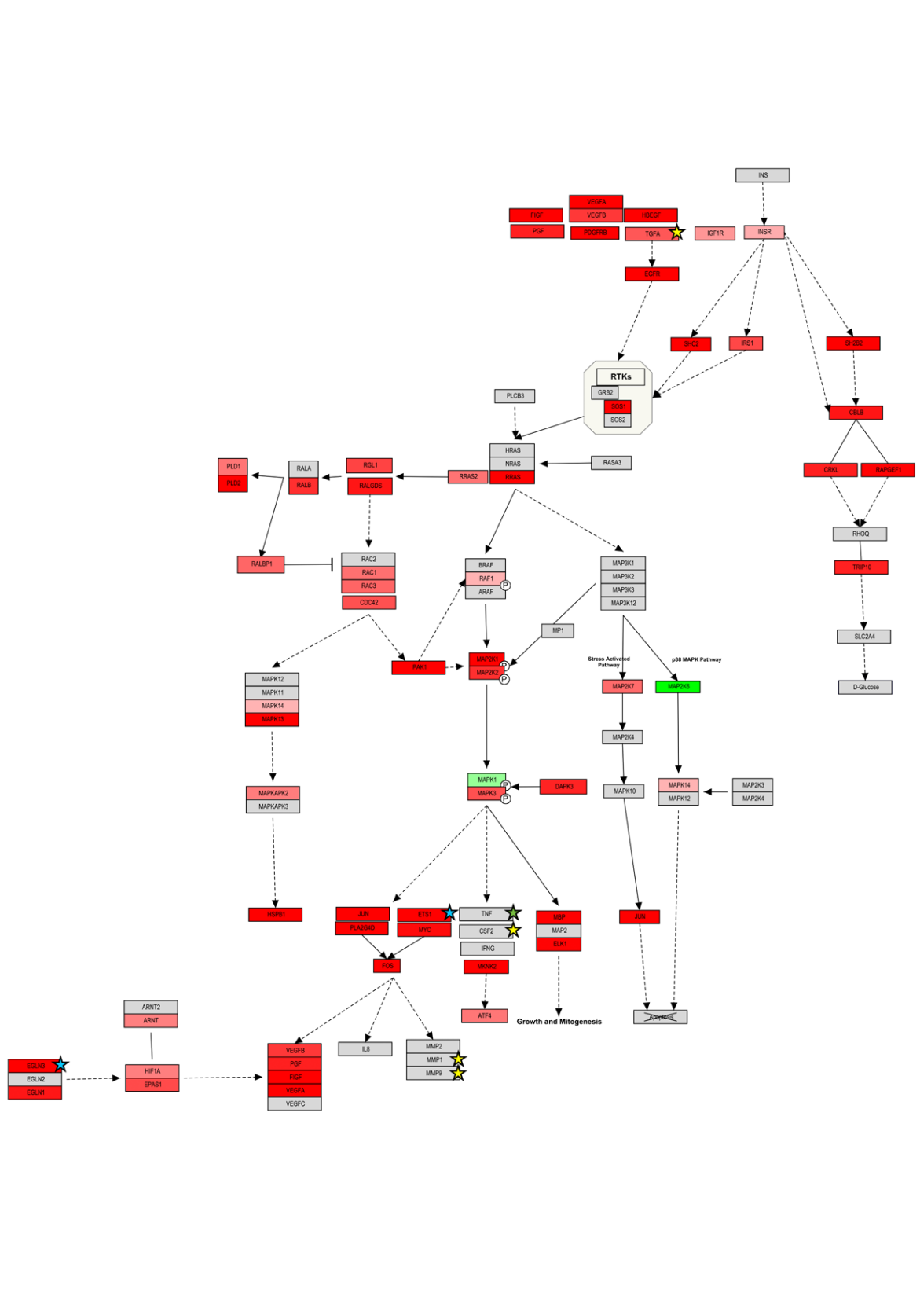
**

**Figure S5.** **Pathway complementation analysis highlights regulatory miRNAs.** a) miR-335 and its gene interactions. b) table of top 10 miRNAs in the miRNA-gene network of group 5 and literature references [1–27].


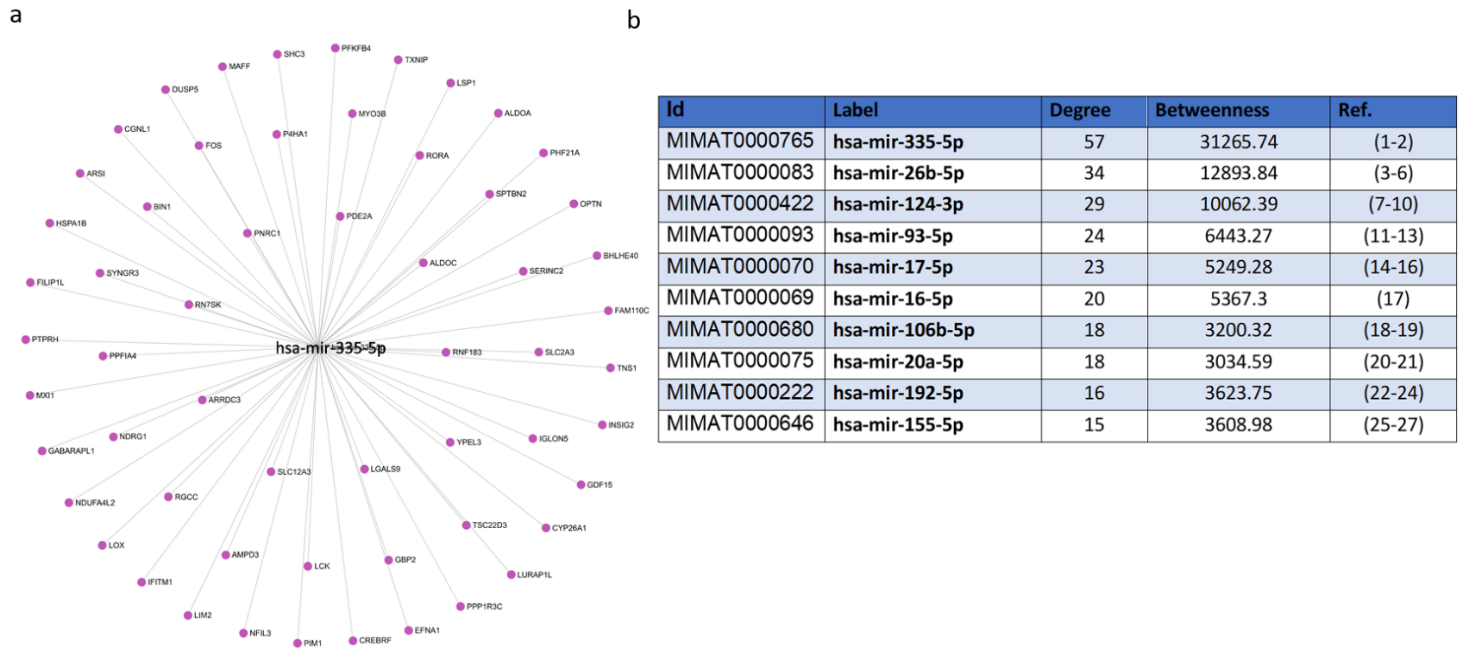
**References**

[1] Wang F, Li L, Piontek K, Sakaguchi M, Selaru FM. Exosome miR-335 as a novel therapeutic strategy in hepatocellular carcinoma. Hepatology 2018;67:940–54. https://doi.org/10.1002/hep.29586.

[2] Cui L, Hu Y, Bai B, Zhang S. Serum miR-335 Level is Associated with the Treatment Response to Trans-Arterial Chemoembolization and Prognosis in Patients with Hepatocellular Carcinoma. Cell Physiol Biochem 2015;37:276–83. https://doi.org/10.1159/000430352.

[3] Sohn W, Kim J, Kang SH, Yang SR, Cho JY, Cho HC, et al. Serum exosomal microRNAs as novel biomarkers for hepatocellular carcinoma. Exp Mol Med 2015;47. https://doi.org/10.1038/emm.2015.68.

[4] Wang X, Liao Z, Bai Z, He Y, Duan J, Wei L. MiR-93-5p promotes cell proliferation through down-regulating PPARGC1A in hepatocellular carcinoma cells by bioinformatics analysis and experimental verification. Genes (Basel) 2018;9. https://doi.org/10.3390/genes9010051.

[5] Shi X, Liu TT, Yu XN, Balakrishnan A, Zhu HR, Guo HY, et al. microRNA-93-5p promotes hepatocellular carcinoma progression via a microRNA-93-5p/MAP3K2/c-Jun positive feedback circuit. Oncogene 2020;39:5768–81. https://doi.org/10.1038/s41388-020-01401-0.

[6] Shan SW, Fang L, Shatseva T, Rutnam ZJ, Yang X, Du WW, et al. Mature miR-17-5p and passenger miR-17-3p induce hepatocellular carcinoma by targeting PTEN, GalNT7 and vimentin in different signal pathways. J Cell Sci 2013;126:1517–30. https://doi.org/10.1242/jcs.122895.

[7] Yang F, Yin Y, Wang F, Wang Y, Zhang L, Tang Y, et al. miR-17-5p promotes migration of human hepatocellular carcinoma cells through the p38 mitogen-activated protein kinase-heat shock protein 27 pathway. Hepatology 2010;51:1614–23. https://doi.org/10.1002/hep.23566.

[8] Awan FM, Naz A, Obaid A, Ikram A, Ali A, Ahmad J, et al. MicroRNA pharmacogenomics based integrated model of miR-17-92 cluster in sorafenib resistant HCC cells reveals a strategy to forestall drug resistance. Sci Rep 2017;7:1–21. https://doi.org/10.1038/s41598-017-11943-1.

[9] Cheng B, Ding F, Huang CY, Xiao H, Fei FY, Li J. Role of miR-16-5p in the proliferation and metastasis of hepatocellular carcinoma. Eur Rev Med Pharmacol Sci 2019;23:137–45. https://doi.org/10.26355/eurrev_201901_16757.

[10] Yu LX, Zhang BL, Yang MY, Liu H, Xiao CH, Zhang SG, et al. MicroRNA-106b-5p promotes hepatocellular carcinoma development via modulating FOG2. Onco Targets Ther 2019;12:5639–47. https://doi.org/10.2147/OTT.S203382.

[11] Li Y, Tan W, Neo TWL, Aung MO, Wasser S, Lim SG, et al. Role of the miR-106b-25 microRNA cluster in hepatocellular carcinoma. Cancer Sci 2009;100:1234–42. https://doi.org/10.1111/j.1349-7006.2009.01164.x.

[12] Chen Y, Wang X, Cheng J, Wang Z, Jiang T, Hou N, et al. MicroRNA-20a-5p targets RUNX3 to regulate proliferation and migration of human hepatocellular cancer cells. Oncol Rep 2016;36:3379–86. https://doi.org/10.3892/or.2016.5144.

[13] H L, H G, F L, J L. Identification of the ASPM-miR-26b-5p network associated with the aggressive traits of HCC cells 2020. https://doi.org/10.21203/RS.3.RS-86573/V1.

[14] Wen Y, Han J, Chen J, Dong J, Xia Y, Liu J, et al. Plasma miRNAs as early biomarkers for detecting hepatocellular carcinoma. Int J Cancer 2015;137:1679–90. https://doi.org/10.1002/ijc.29544.

[15] Tan Y, Lin B, Ye Y, Wen D, Chen L, Zhou X. Differential expression of serum microRNAs in cirrhosis that evolve into hepatocellular carcinoma related to hepatitis B virus. Oncol Rep 2015;33:2863–70. https://doi.org/10.3892/or.2015.3924.

[16] Gu Y, Wei X, Sun Y, Gao H, Zheng X, Wong LL, et al. MiR-192-5p silencing by genetic aberrations is a key event in hepatocellular carcinomas with cancer stem cell features. Cancer Res 2019;79:941–53. https://doi.org/10.1158/0008-5472.CAN-18-1675.

[17] Gu Y, Ji F, Liu N, Zhao Y, Wei X, Hu S, et al. Loss of miR-192-5p initiates a hyperglycolysis and stemness positive feedback in hepatocellular carcinoma. J Exp Clin Cancer Res 2020;39:1–17. https://doi.org/10.1186/s13046-020-01785-7.

[18] Yao Y, Shu F, Wang F, Wang X, Guo Z, Wang H, et al. Long noncoding RNA LINC01189 is associated with HCV-hepatocellular carcinoma and regulates cancer cell proliferation and chemoresistance through hsa-miR-155-5p. Ann Hepatol 2021;22. https://doi.org/10.1016/j.aohep.2020.09.013.

[19] Yerukala Sathipati S, Ho SY. Novel miRNA signature for predicting the stage of hepatocellular carcinoma. Sci Rep 2020;10:14452. https://doi.org/10.1038/s41598-020-71324-z.

[20] Chen G, Wang D, Zhao X, Cao J, Zhao Y, Wang F, et al. MiR-155-5p modulates malignant behaviors of hepatocellular carcinoma by directly targeting CTHRC1 and indirectly regulating GSK-3β-involved Wnt/β-catenin signaling. Cancer Cell Int 2017;17:118. https://doi.org/10.1186/s12935-017-0469-8.

[21] Miyamoto K, Seki N, Matsushita R, Yonemori M, Yoshino H, Nakagawa M, et al. Tumour-suppressive miRNA-26a-5p and miR-26b-5p inhibit cell aggressiveness by regulating PLOD2 in bladder cancer. Br J Cancer 2016;115:354–63. https://doi.org/10.1038/bjc.2016.179.

[22] Khosla R, Hemati H, Rastogi A, Ramakrishna G, Sarin SK, Trehanpati N. miR-26b-5p helps in EpCAM+cancer stem cells maintenance via HSC71/HSPA8 and augments malignant features in HCC. Liver Int 2019;39:1692–703. https://doi.org/10.1111/liv.14188.

[23] Wang Y, Sun B, Sun H, Zhao X, Wang X, Zhao N, et al. Regulation of proliferation, angiogenesis and apoptosis in hepatocellular carcinoma by miR-26b-5p. Tumor Biol 2016;37:10965–79. https://doi.org/10.1007/s13277-016-4964-7.

[24] Long HD, Ma YS, Yang HQ, Xue SB, Liu J Bin, Yu F, et al. Reduced hsa-miR-124-3p levels are associated with the poor survival of patients with hepatocellular carcinoma. Mol Biol Rep 2018;45:2615–23. https://doi.org/10.1007/s11033-018-4431-1.

[25] Majid A, Wang J, Nawaz M, Abdul S, Ayesha M, Guo C, et al. miR-124-3p Suppresses the Invasiveness and Metastasis of Hepatocarcinoma Cells via Targeting CRKL. Front Mol Biosci 2020;7. https://doi.org/10.3389/fmolb.2020.00223.

[26] Zhong D, Lyu X, Fu X, Xie P, Liu M, He F, et al. Upregulation of miR-124-3p by Liver X Receptor Inhibits the Growth of Hepatocellular Carcinoma Cells Via Suppressing Cyclin D1 and CDK6. Technol Cancer Res Treat 2020;19. https://doi.org/10.1177/1533033820967473.

[27] Wu LP, Wu J, Shang A, Yang M, Li LL, Yu J, et al. miR-124 inhibits progression of hepatocarcinoma by targeting KLF4 and promises a novel diagnostic marker. Artif Cells, Nanomedicine Biotechnol 2018;46:159–67. https://doi.org/10.1080/21691401.2017.1415918.
